# Characterizing CD44 regulatory microRNAs as putative therapeutic agents in human melanoma

**DOI:** 10.18632/oncotarget.27305

**Published:** 2019-11-05

**Authors:** Johannes Fänder, Heike Kielstein, Maximilian Büttner, Peter Koelblinger, Reinhard Dummer, Marcus Bauer, Diana Handke, Claudia Wickenhauser, Barbara Seliger, Simon Jasinski-Bergner

**Affiliations:** ^1^Institute of Anatomy and Cell Biology, Faculty of Medicine, Martin Luther University Halle-Wittenberg, Halle, Germany; ^2^Department of Dermatology, Paracelsus Medical University, Salzburg, Austria; ^3^Department of Dermatology, University Hospital of Zurich, Zurich, Switzerland; ^4^Institute for Pathology, Faculty of Medicine, Martin Luther University Halle-Wittenberg, Halle, Germany; ^5^Institute for Medical Immunology, Faculty of Medicine, Martin Luther University Halle-Wittenberg, Halle, Germany

**Keywords:** CD44, CD44v6, melanoma, microRNA, immune cell infiltration

## Abstract

The multistructural and multifunctional transmembrane glycoprotein CD44 is overexpressed in many tumors of distinct origin including malignant melanoma and contributes to a poor prognosis by affecting cell proliferation, cell migration, and also the sensitivity for apoptosis induction. Previous studies reported so far 15 CD44 regulatory microRNAs (miRs) in different cell systems.

Using a novel method for miR affinity purification miR-143-3p was identified as most potent binder to the 3’ untranslated region (UTR) of CD44. Overexpression of miR-143-3p in melanoma cells inhibits CD44 translation, which is accompanied by a reduced proliferation, migration and enhanced daunorubicin induced apoptosis of melanoma cells *in vitro*.

Analyses of discordant CD44 and miR-143-3p expression levels in human melanocytic nevi and dermal melanoma samples demonstrated medium to high CD44 levels with no association to tumor grading or staging. The CD44 expression correlated to PD-L1, but not to MART-1 expression in malignant melanoma. Interestingly, the CD44 expression was inversely correlated to the infiltration of pro-inflammatory immune effector cells.

In conclusion, the tumor suppressive miR-143-3p was identified as the most potent CD44 inhibitory miR, which affects growth characteristics of melanoma cells suggesting the implementation of miR-143-3p as as a potential anti-CD44 therapy of malignant melanoma.

## Introduction

The transmembrane glycoprotein CD44 is constitutively expressed on embryonic stem cells, in connective tissues and in the bone marrow and overexpressed in different tumor entities as well as in cancer stem cells (CSC). Upon binding to its ligand, the extracellular matrix (ECM) component hyaluronic acid (HA), CD44 plays a key role in various physiological functions by activating different signal transduction pathways leading to cell proliferation, adhesion, migration, angiogenesis and inflammation [1–3], but also in pathophysiologic processes, such as self-renewal, tumor initiation, epithelial mesenchymal transition, metastasis and chemoresistance [4, 5]. Furthermore, CD44 has been suggested as a diagnostic marker for CSC and as a prognostic marker for different tumor entities correlating with a poor prognosis of tumor patients [4, 6].

The human CD44 gene is highly conserved, localized on the short arm of chromosome 11 and encoded by 20 exons, from which 10 are constant in all isoforms [7]. The “standard” CD44 isoform (CD44s) encoded by the 10 constant exons has a molecular weight of 85–90 kDa and is ubiquitously expressed in vertebrates. The “variant” isoforms (CD44v) are generated by alternative splicing and comprise next to the 10 constant exons any combination of the remaining variant exons forming 42 currently known CD44 isoforms at the mRNA level, of which 29 encode for proteins [8, 9]. In contrast to CD44s, the distribution of CD44v isoforms is restricted to selected epithelial, proliferating and even tumor cells [2, 10, 11].

The diversity of the CD44 protein family is mediated by alternative splicing, and by posttranslational modifications, including massive glycosylation and domain cleavage. These properties highly increase the functional variety and binding capacity of CD44 [4]. In addition, the CD44 gene expression is post-transcriptionally regulated by microRNAs (miRs), which are small, highly conserved, non-coding RNAs that preferentially bind to the 3‘untranslated region (UTR) of the target mRNA leading to mRNA decay and translational inhibition [12]. So far, at least 15 different CD44-regulating miRs have been identified mainly exhibiting tumor suppressive activities by exerting anti-proliferative, anti-invasive, anti-angiogenic and apoptosis-promoting functions (Table 1; [13, 14, 23–26, 15–22]).

**Table 1:** Published CD44 regulatory miRs and their tumor biological effects

tumor biological effect of the miR					
**miRs**	**proliferation**	**invasion**	**apoptosis**	**angiogenesis**	**tumor suppressive/oncogenic**
**miR-143-3p**	↓	↓	↑	↓	tumor suppressive
**miR-199a-3p**	↓	↓	↑	↓	tumor suppressive
miR-216a	↓	↓	↑	↓	tumor suppressive
miR-328	↓	↓	↑	↓	tumor suppressive
miR-330	↓	↓	↑	n.r.	tumor suppressive
**miR-34a-5p**	↓	↓	↑	↓	tumor suppressive
miR-373	controversial discussed				
**miR-491-5p**	↓	↓	↑	↓	tumor suppressive
miR-492	↑	↑	n.r.	n.r.	oncogenic
miR-512-3p	↓	↓	↑	n.r.	tumor suppressive
miR-520c	controversial discussed				
miR-541-3p	n.r.	n.r.	↓	n.r.	oncogenic
miR-608	↓	↓	↑	n.r.	tumor suppressive
miR-671	n.r.	n.r.	n.r.	n.r.	n.r.
miR-708	controversial discussed				

Malignant melanoma (MM) is one of the most aggressive neoplasms derived from malignant transformation of melanocytes with an increasing incidence worldwide over the decades. The main risk factors include exposure to ultraviolet (UV) light, genetic susceptibility and the number of melanocytic nevi [47, 48]. With the introduction of immunotherapy and targeted therapies the outcome of patients increased due to response rates of 20–40% [49]. However, there is still an urgent need for biomarkers to distinguish therapy responders from non-responders and also of novel therapeutic targets.

While melanocytes only express CD44s, benign melanocytic lesions, melanoma lesions and melanoma cell lines heterogeneously express both CD44s as well as CD44v isoforms [27, 28]. Interestingly, patients with higher (pan)CD44 expression had a significant reduced 5-years’ overall survival (OS). Targeting of CD44 in experimental models *in vivo* resulted in an increased survival rate of mice [29]. Investigations regarding the potential of CD44 as therapeutic target used multiple approaches, including monoclonal antibodies (mAb) [30], peptides like A5G27 or PEX9 [31, 32] and post-transcriptional down regulation by RNAi [33]. Currently, miRs are of growing interest, not only as diagnostic, prognostic or predictive biomarkers, but also as therapeutic tools. So far, 15 human CD44 regulatory miRs have been identified in various cell systems (Table 1), but their functional relevance has not yet been determined. Therefore, these miRs were characterized regarding their binding affinity to the 3’ UTR of CD44s, their tumor biological functions and their potential as putative miR-based anti-cancer drug in melanoma.

## Results

**Figure 1:**
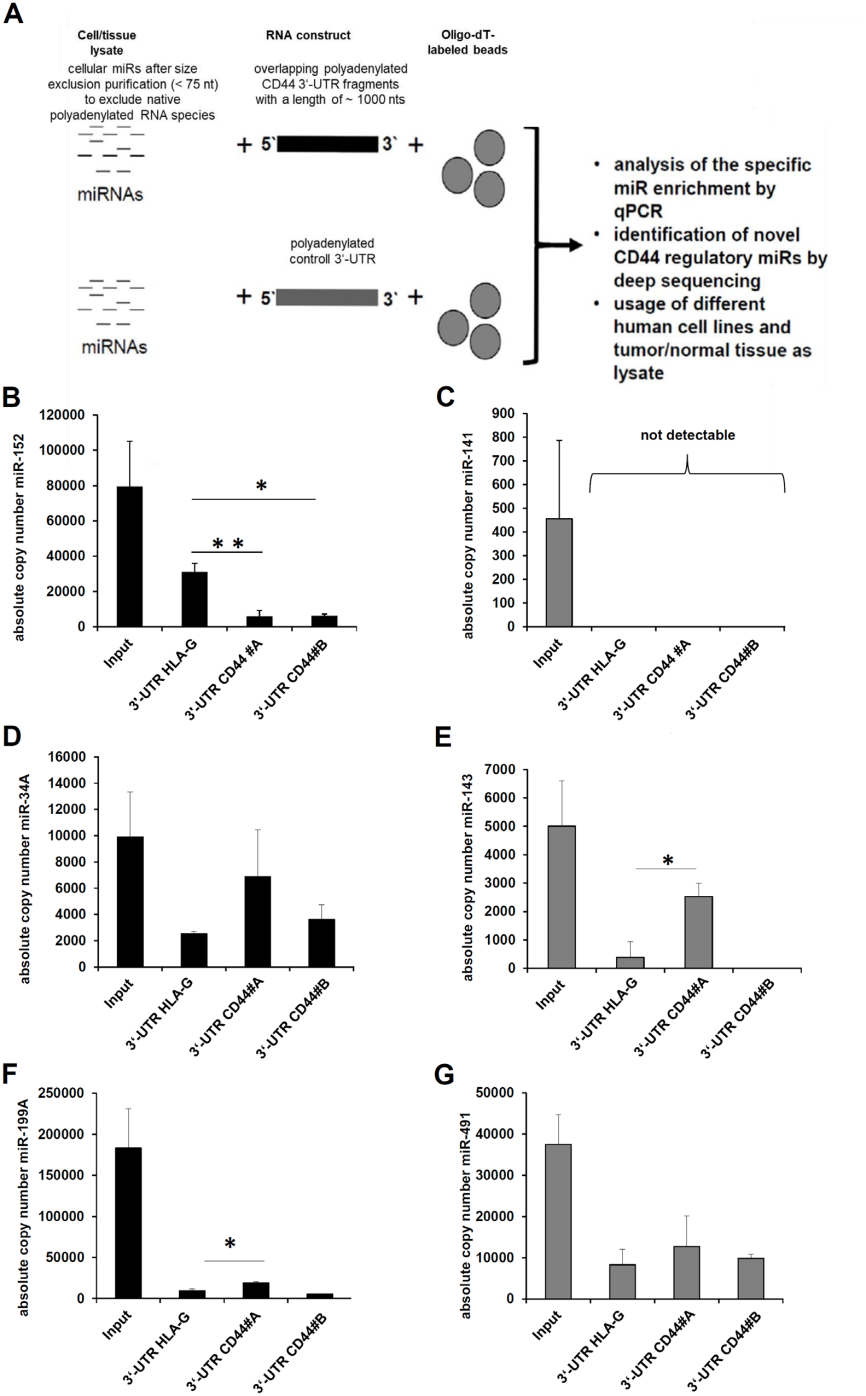
Establishment of a novel affinity based miR enrichment assay. (**A**) The workflow of the applied novel miR affinity purification assay is summarized. (**B–G**) The eluates and the respective amount of cell lysate (input) were analyzed by qPCR as described in Materials and Methods. As internal positive control the enrichment of miR-152 with the HLA-G 3’ UTR as bait was determined (B) as well as negative control (C) the absent enrichment of miR-141, which was detected in the applied cell lysates (input). Furthermore, the exemplary validation of the known CD44 regulatory miRs; miR-34-5p (D), miR-143-3p (E), miR-199A-3p, and miR-491-5p (G) is shown.

Despite more than 15 CD44 regulatory miRs have been reported in the literature (Table 1). There exists so far no information about their affinity to the CD44 3’ UTR in general and in particular for melanoma cells. Therefore, the affinity of these miRs was validated and compared to the reported binding sites within the CD44 3’ UTR by using a novel miR-specific enrichment assay (Figure 1A).

The eluates of this affinity purification were analyzed by qPCR. The interaction between the HLA-G 3’ UTR and miR-152 served as positive control [38]. As expected, miR-152 was statistically significant enriched with the HLA-G 3’ UTR as bait, but not with the two fragments (#A and #B) of the CD44 3’ UTR (Figure 1B). MiR-141 as a negative control was present in the applied cell lysate (input), but neither enriched with the HLA-G 3’ UTR nor with the CD44 3’ UTR (Figure 1C). Concerning the affinity purification of the known CD44 regulatory miRs, miR-34A-5p and miR-143-3p were highly enriched with the CD44 3’ UTR as bait when compared to the HLA-G 3’ UTR, which was statistically significant for miR-143-3p (Figure 1D–1E). The miR-199A and miR-491 were also enriched, but to a weaker extent (Figure 1F–1G), while the other reported CD44 regulatory miRs were not enriched with the reported CD44 3’ UTR fragment. However, an interaction of these miRs with the coding sequence or the 5’ UTR of the CD44 mRNA cannot be excluded. Despite these miRs were published as binding to the investigated CD44 3’ UTR, they were excluded from further experiments. The miR-541-3p was not expressed in the applied cell lysate.

**Figure 2:**
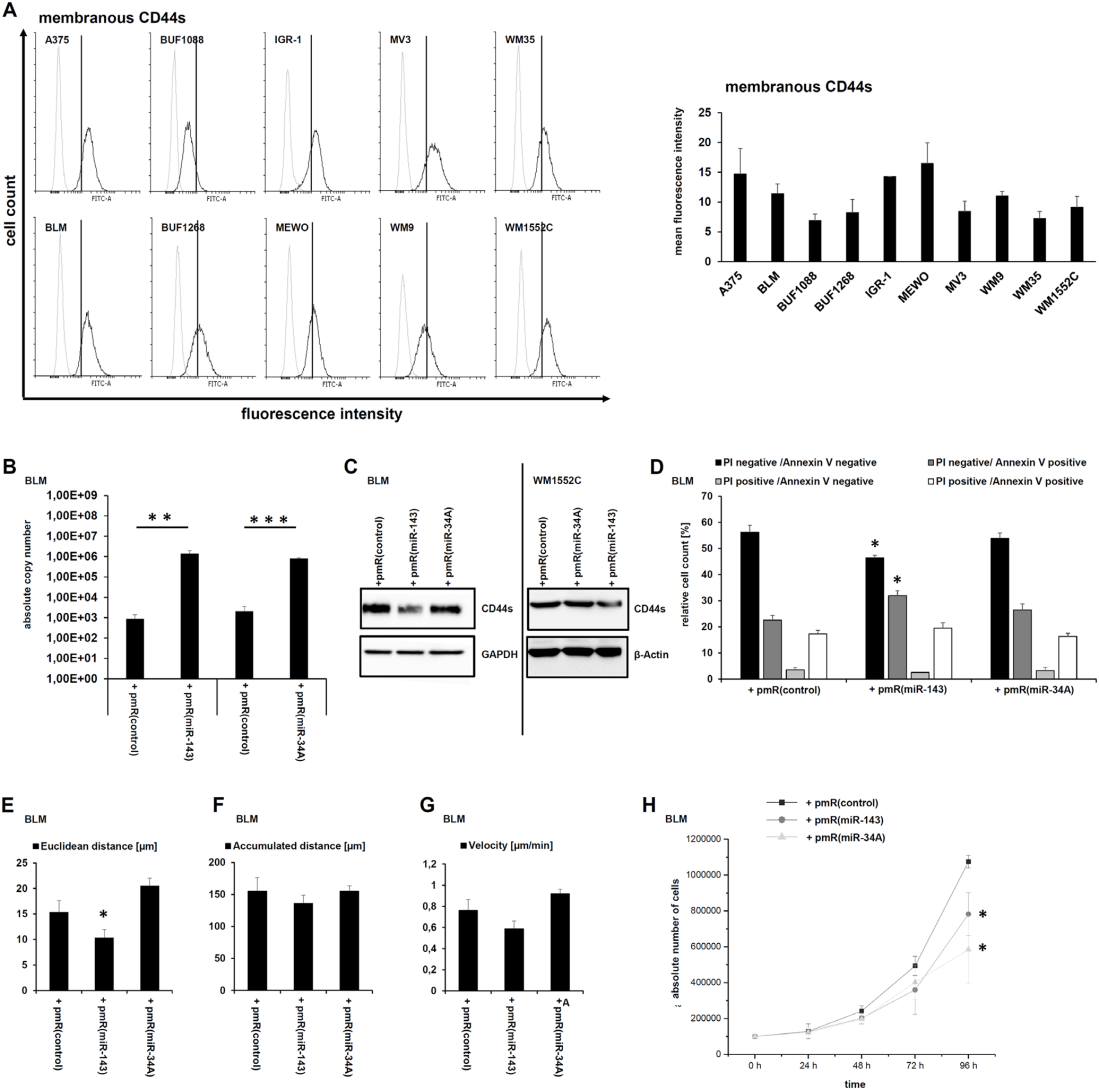
Modulation of tumor biological relevant functions by miR-143-3p-mediated CD44s downregulation. (**A**) The membranous CD44s expression of 10 human melanoma cell lines was quantified by flow cytometry and exemplary visualized for one of three biological replicates as overlay. The grey histograms represent the isotype control and the black histogram staining with the specific CD44s antibody as described in Materials and Methods. (**B**) The functionality of the miR expression vectors was validated by qPCR after transient transfection in the human melanoma cell line BLM as described in Materials and Methods. (**C**) Western blot analyses of transiently miR-143-3p transfected BLM cells as well as of stably transfected WM1552C cells were performed using a CD44 specific antibody. (**D**) Flow cytometry based Annexin V and propidium iodide (PI) staining for daunorubicin mediated apoptosis in the miR-143-3p transfectants was performed. (E–H) Cell migration, in particular euclidean distance (**E**), accumulated distance (**F**), and velocity (**G**) as well as proliferation (**H**) was analyzed in the transfectants.

Ten melanoma cell lines were investigated for their CD44s expression using flow cytometry (Figure 2A). The BLM, WM1552C, and A375 cell lines exerted the highest CD44s expression levels. Due to the high transfection efficacy the BLM cell line was selected for further analyses. MiR-34A-5p and miR-143-3p overexpression in BLM cells was validated by qPCR demonstrating a statistically significant miR overexpression of a three-digit factor when compared to the mock vector (Figure 2B). The strong overexpression of miR-143-3p led to a reduction of total CD44s protein in transient transfected BLM cells as well as in stable transfected WM1552C cells, while for miR-34A no reduction of CD44s protein was observed (Figure 2C).

To determine whether the miR based CD44s downregulation affects the sensitivity of melanoma to the therapy-relevant drug daunorubicin, the cell viability of untreated and daunorubicin-treated miR transfectants was determined. The reduced CD44s protein levels upon miR-143-3p overexpression caused a statistically significant reduction of viable cells (propidium iodide (PI)/Annexin V double negative) and a statistically significant increase of early apoptotic cells (PI negative/Annexin V positive). The amount of late apoptotic cells (PI/Annexin V double positive) was also increased, but not statistically significant (Figure 2D).

Furthermore, the miR-143-3p mediated CD44s downregulation also affected cell migration. Actually, the velocity [µm/min], the euclidean distance [µm] and the total accumulated distance [µm] were strongly reduced in the miR-143-3p overexpressing transfectants when compared to the other transfectants (Figure 2E–2G). Since miR-143-3p as well as miR-34A-5p reduced proliferation (Table 1), the proliferation of the transfectants was characterized. As shown in Figure 2H, both miR overexpressing transfectants exerted a statistically significant reduced proliferation rate in comparison to mock transfectants (Figure 2H).

**Figure 3:**
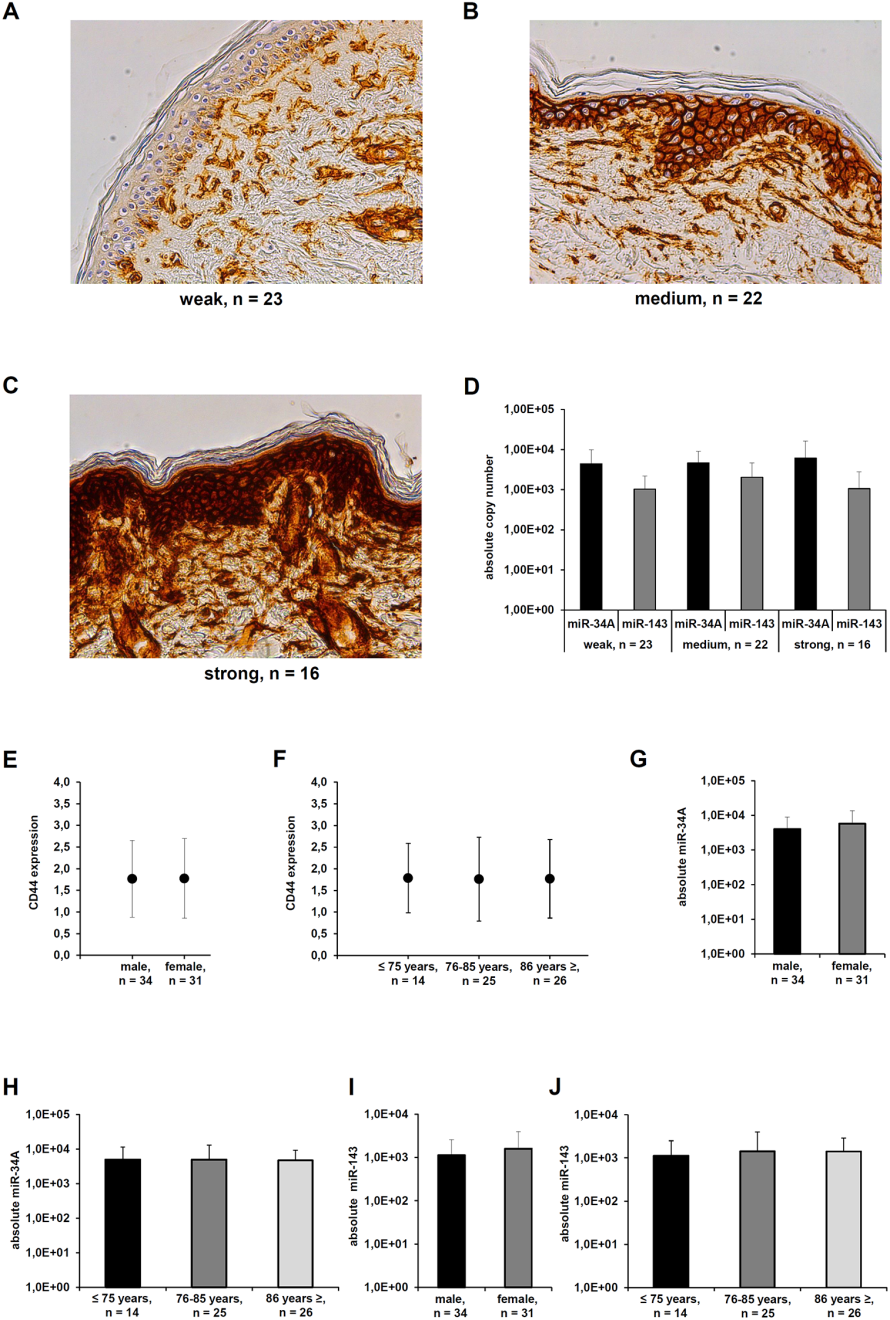
Differential CD44s expression in healthy skin sections. (**A–C**) A representative immunohistochemical staining of human skin samples derived from human body donors with distinct CD44s expression shown. The staining was scored as weak, medium, and strong expression. (**D**) qPCR analyses of human skin samples with distinct CD44s expression levels was used to determine the absolute miR-34a-5p and miR-143 absolute copy number as described in Materials and Methods. (**E–J**) The expression levels of CD44s protein (E, F), miR-34A-5p (G, H), and miR-143-3p (I, J) were correlated to selected clinical parameters from the human body donors.

The *in vivo* CD44s expression was also analyzed in non-affected skin sections (gluteal region) derived from 61 human body donors by immunohistochemistry (IHC). CD44s expression was observed in the whole stratum germinativum, consisting of the stratum basale and the stratum spinosum. The stratum granulosum and stratum corneum were unreactive, which is in line with already published studies [40]. CD44s positive cells accumulated in the proliferative epidermis regions. The CD44s expression of the epidermal melanocytes was comparable to that of dermal keratinocytes ranging from weak (23/61), over medium (22/61) to strong (16/61) expression. Interestingly, the CD44s expression appears to correlate with the thickness of the stratum basale. Occasional CD44s positive spots were also visible within the dermis with a higher frequency within the stratum papillare than within the stratum reticulare (Figure 3A–3C). However, there existed no inverse correlation between the *in vivo* CD44s protein levels and the expression of miR-143-3p and miR-34A-5p determined by qPCR (Figure 3D). The expression levels of CD44s protein and miR-34A-5 as well as miR-143-3p were further correlated to clinical data of the investigated human body donors (Figure 3E–3J).

**Figure 4:**
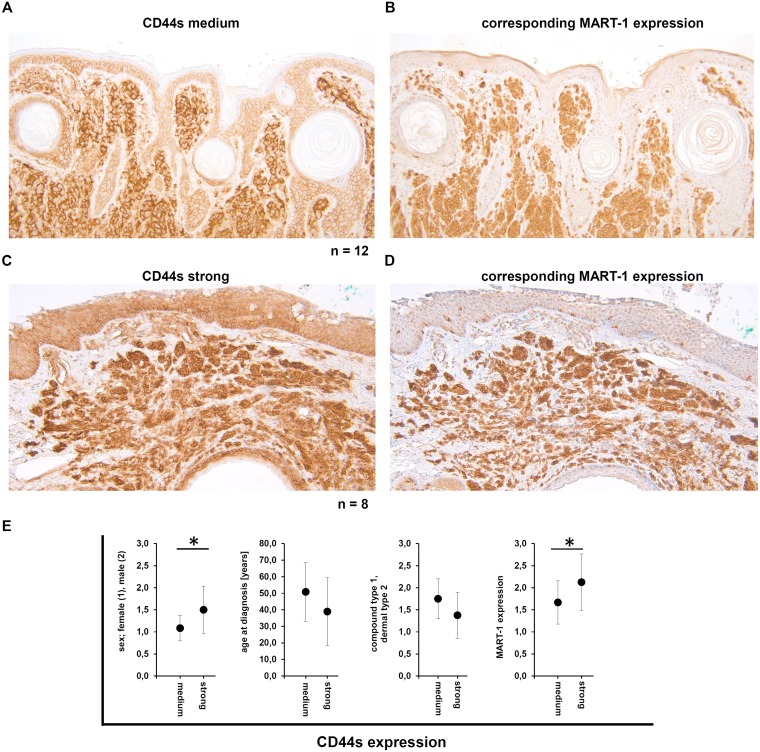
Demonstration of the CD44s and the corresponding MART-1 expression in human melanocytic nevi. (**A–D**) Immunohistochemical staining for CD44s demonstrated either medium (A) or high (C) CD44s expression in the melanocytic nevi, as well as the corresponding MART-1 expression. (**E**) Correlation of the CD44s expression level with clinical parameters, including sex, age at diagnosis, type of nevi (compound type or dermal), and the correlation to the MART-1 expression.

As a cell type specific CD44s expression was also analyzed in the context of benign melanocytic lesions (Table 2). Evaluating diverse subtypes of melanocytic nevi, CD44s expression levels ranged from medium to high. In detail, in the compound nevi type the CD44s expression was higher than in the deeper dermis localized dermal nevi type, but this effect was not statistically significant. In addition, the group of nevi with strong CD44s expression showed a lower mean age at diagnosis and a statistically significant higher amount of male patients (Figure 4E). When compared to the malignant melanoma probes, only in melanocytic nevi a statistically significant correlation between MART-1 and CD44s expression was detected (Figure 4A–4E).

**Table 2:** Melanocytic nevi patient’s characteristics

patient no.	age at diagnosis [years]	sex	type of nevus
1	13	male	compound
2	51	male	compound
3	72	female	dermal
4	72	female	dermal
5	41	female	dermal
6	76	female	dermal
7	27	female	dermal
8	27	female	compound
9	55	female	dermal
10	45	female	dermal
11	81	female	dermal
12	37	female	dermal
13	37	female	dermal
14	44	female	compound
15	13	male	dermal
16	44	female	compound
17	42	male	compound
18	59	female	compound
19	31	female	compound
20	54	male	dermal

**Figure 5:**
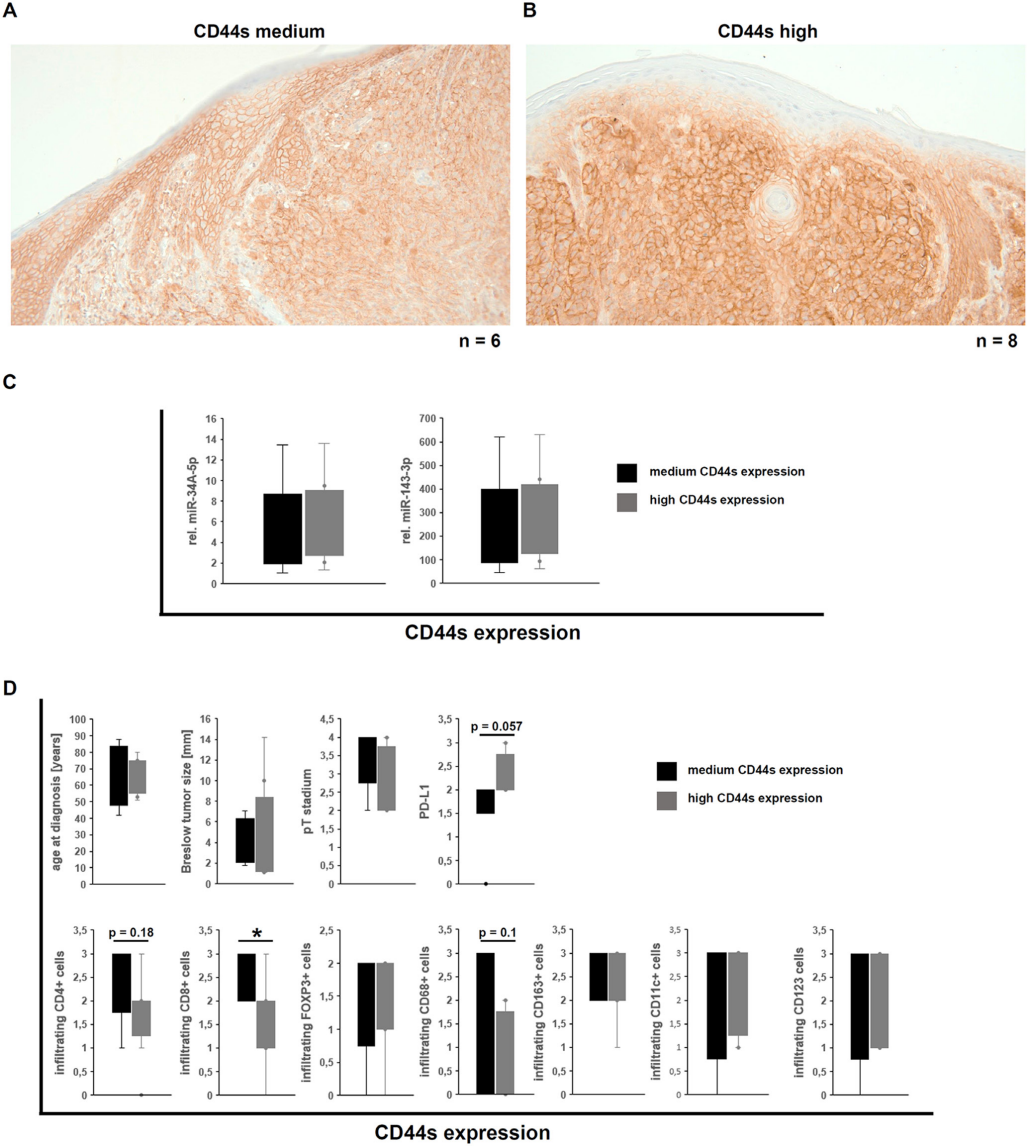
Correlation of the CD44s expression in human malignant melanoma with miR expression and immune cell infiltration and its tumor biological impact. (**A–B**) Immunohistochemical staining for CD44s demonstrated either medium (A) or high (B) CD44s expression in the malignant melanoma samples. (**C**) Correlation of miR-34A-5p and miR-143-3p with the CD44s protein levels in malignant melanoma samples. (**D**) Correlation of CD44s expression levels to clinical parameters from the melanoma patients and to infiltrating immune effector cells [34].

**Figure 6:**
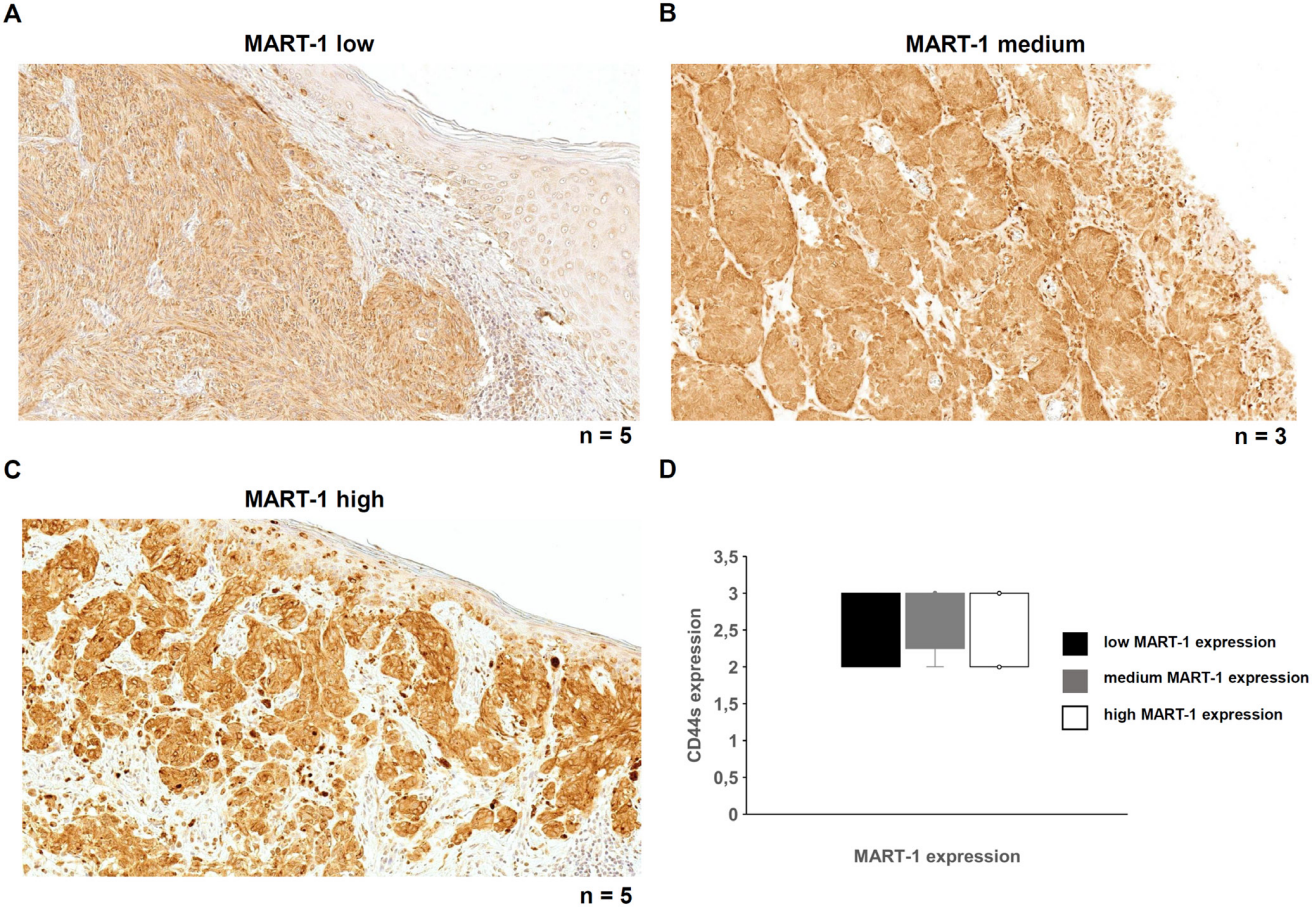
MART-1 expression and its correlation with CD44s expression in malignant melanoma. (**A–C**) Immunohistochemical analysis was performed with a MART-1 specific mAb to determine MART-1 expression in human malignant melanoma samples. A heterogeneous MART-1 expression was found ranging from low (A), medium (B) or high (C) MART-1 expression in all investigated melanoma samples. (**D**) MART-1 and CD44s expression levels were correlated.

The CD44s expression in human melanoma samples was correlated with known clinical characteristics (Table 3). All investigated tumors exerted medium to high CD44s expression. Tumors with a medium CD44s expression level showed a more cytoplasmic localization, while tumors with high CD44s levels presented an additional membranous expression. In the adjacent epidermis, the CD44s expression was detected in cells of the stratum germinativum, but not within the stratum granulosum and stratum corneum, while cells in the dermis were also stained. In all cases analyzed the CD44 expression was stronger in melanoma cells when compared to the adjacent epidermal cells (Figure 5A–5B). In analogy to the skin samples of the human body donors as well as the benign melanocytic lesions, the CD44s protein and the miR-34A-5p expression levels as well as miR-143-3p expression levels showed no inverse correlation (Figure 5C). For a better visualization of the melanoma cells, MART-1 expression was determined by immunohistochemistry. MART-1 expression levels in the malignant melanoma ranged from low to high (Figure 6A–6C) without any correlation between the MART-1 and CD44s expression levels (Figure 6D).

**Table 3:** Malignant melanoma patient’s characteristics

patient no.	age at diagnosis [years]	sex	ulcerated	Breslow [mm]	pT stadium	type of melanoma	recurrence
1	71	male	no	2.15	3	superficial spreading melanoma	no
2	62	female	yes	1.1	2	superficial spreading melanoma	no
3	82	male	yes	3.5	3	nodular melanoma	yes
4	50	male	yes	2.6	3	nodular melanoma	no
5	64	male	no	3.2	3	superficial spreading melanoma	no
6	51	female	no	1.11	2	superficial spreading melanoma	no
7	88	female	no	7.05	4	nodular melanoma	no
8	75	male	no	1.3	2	superficial spreading melanoma	no
9	80	male	yes	3.5	3	nodular melanoma	no
10	64	female	no	10	4	nodular melanoma	unknown
11	42	female	yes	6.1	4	nodular melanoma	no
12	53	male	yes	14.2	4	nodular melanoma	yes
13	59	male	no	1.8	2	superficial spreading melanoma	no
14	75	female	yes	3.08	3	nodular melanoma	yes

Based on the recently described immune cell infiltration and marker analysis [34], an almost statistically significant (*p* = 0.057) correlation of CD44s expression with PD-L1 (B7-H1) was found in the MM samples analyzed, while partially statistically significant inverse correlation between CD44s protein levels and infiltrating immune effector cells, like CD4^+^ cells (*p* = 0.18), CD8^+^ cells (*p* = 0.03), and CD68^+^ M1 macrophages (*p* = 0.1) was observed. There existed no correlation between CD44s protein levels and the infiltrating regulatory FOXP3^+^ T cells, M2 macrophages (CD123), and dendritic cells (CD11c and CD123; Figure 5D). Furthermore, no effect of the CD44s protein levels, the tumor thickness (Breslow value) and the pT stadium could be observed (Figure 5D).

## Discussion

During the last years, the number of publications reporting miR target interactions is steadily increasing. Often, studies describe many different miRs against one certain target mRNA in complete different cell systems under varying conditions with marginally comparable methods. There, the role of known CD44-regulatory miRs was analyzed in melanoma. The inhibition of CD44 using anti-CD44 antibodies as anti-melanoma treatment showed success in an experimental study [41], while a recent phase I clinical trial of an anti-CD44 humanized antibody using the RG7356 in patients with metastatic or locally advanced CD44-expressing solid malignancies (6 melanoma from 65 different solid tumors of various entities) only demonstrated a modest anti-tumor activity [42] demanding alternative strategies to overcome CD44 expression in tumors. In this context, a miR-based down regulation of CD44 might be a better therapeutic tool. This approach extends the activity of all CD44 splice variants, since the 3’ UTR of CD44 splice variants is equal. In contrast, therapeutic antibodies potentially do not bind to all of the different CD44 splice variants and thus their binding may not completely inhibit the biological function of CD44.

First, the affinity of the 15 known miRNAs binding to the CD44 3’ UTR was determined using a novel modified miTRAP technique. While the original miTRAP technology is based on interaction between MS2 tagging and a not commercial-available fusion protein containing the MS2 loop binding protein and the maltose binding protein, the *in vitro* transcribed 3’ UTRs were not tagged with MS2 loops and loaded out amylose beads, but were polyadenylated and coupled to commercial-available oligo dT coated beads. To avoid the binding of polyadenylated mRNA species also present in the cell lysate, the lysates were purified prior usage by size exclusion columns (≤ 75 nt). This miR containing flow through was used for later affinity purification. The functionality of this modified miTRAP technique was validated by the enrichment of the miR-152 with the HLA-G 3’ UTR as bait (positive control), while the miR-141 was present in the applied cell lysate (input), but neither enriched (negative control; [43] with the HLA-G 3’ UTR nor with the CD44 3’ UTR.

Four of 15 CD44-regulatory miRs were enriched using the CD44 3’ UTR as bait, which due to its size was divided into two overlapping fragments. With respect to their abundance in the cell lysate (input), miR-34A-5p and miR-143-3p exerted the highest affinities for the CD44 3’ UTR. However, only miR-143-3p overexpression caused a downregulation of CD44s protein linked to a decreased proliferation and cell migration as well as an enhanced sensitivity for daunorubicin-induced apoptosis. This is in line with the association of CD44 overexpression with tumor progression and metastasis in various human cancers including melanoma. Furthermore, patients with high CD44 expression levels in malignant melanoma have a statistical significantly decreased 5 years’ OS in comparison to the CD44 low group [29].

In addition, CD44 expression almost statistically significantly correlated with PD-L1 expression and with a trend can inverse correlation to tumor infiltrating pro-inflammatory immune effector cells. Despite the PD-1 checkpoint inhibitor pembrolizumab is used for melanoma therapy [44] and the PD-L1 checkpoint inhibitor atezolizumab is still under investigation in ongoing clinical trials with melanoma patients, the success is limited and patients develop resistances over treatment. Thus, targeting might be a promise therapeutic tool for malignant melanoma.

Due to the fact of high CD44 expression levels in various tumor diseases, anti-CD44 antibodies are currently under investigation as potential anti-tumoral immune therapeutics in different animal studies to reduce tumor progression [45]. However, an antibody based anti-CD44 therapy will face certain limitations such as (i) the existence of different CD44 splicing variants lacking several protein domains and (ii) the fact that CD44 is also expressed in various healthy tissues. In contrast, miR-based CD44 inhibition limited to malignant cells would affect all CD44 splice variants, since they share the same 3’ UTR. Recent studies report the functionality of miRs linked to antibodies directed against cell surface molecules, like membranous tumor antigens and receptors. After binding of the antibody to the tumor antigen on the cell surface, which already blocks its functionality, the following internalization of this complex further enables the miR-induced effects like downregulation of tumor relevant genes [46].

A prerequisite is, however, that only miRs are applied for such strategies, which are able to down-regulate the translation of the gene of interest. Therefore, this study aimed to identify the most potent CD44-regulatore miRs published so far in melanoma with the highest potential for miR-143-3p. However, concluding from the missing inverse correlation of miR-143-3p and CD44s expression in healthy and malignant skin tissues as well as in the investigated melanoma cell lines (data not shown), miR-143-3p is able to inhibit CD44 translation and function upon overexpression, but does not seem to be the key regulating miR in the *in vivo* cellular situation. Hence, the authors hypothesize that other so far unknown CD44 regulatory miRs may exist, which could even exert a stronger potential on the CD44 translational inhibition. Indeed, since the beginning of the present study other CD44-regulating miRs have been reported. These novel miRs have not been considered within the present study. To improve studies identifying single miRs regulating CD44 or even other proteins, the authors suggest to apply the novel miR affinity purification method presented in this study in combination with next generation sequencing to identify a large panel of miRs binding to the 3’ UTR of CD44. These could then be used for further functional studies.

Furthermore the authors want to advise that instead of CD123 as a marker for M2 macrophages the markers CD162 and/or CD203 would be even better markers.

## Materials and Methods

### Cell culture and treatment

The melanoma cell lines A-375, BLM, BUF-1088, BUF-1268, IGR-1, MEVO, MV-3, WM-9, WM-35 and WM-1552C and the CD44 negative human embryonal kidney cell line HEK293T were cultured in Dulbecco’s modified Eagles medium (DMEM; Merck Millipore, Heidelberg, Germany) supplemented with 10% fetal calf serum (FCS; Merck Millipore, Heidelberg, Germany), 1% penicillin/streptomycin (Sigma Aldrich, St.Louis, USA), 1% sodium pyruvate (Merck Millipore, Heidelberg, Germany), 2 mM L-glutamine (Merck Millipore, Heidelberg, Germany) and detached with trypsin (Sigma Aldrich, St.Louis, USA).

### Human samples analyzed

Random human skin tissues were collected from human body donors (*n* = 65) in the Institute of Anatomy and Cell Biology, Martin Luther University Halle-Wittenberg, Halle, Germany from 2015 to 2018. In addition, tissue samples of human melanocytic nevi (*n* = 20) archived between 2010 and 2014 in the Institute for Pathology of the Martin Luther University Halle-Wittenberg were selected as non-malignant melanocytic lesions. Archived tissue samples of cutaneous MM samples collected between 2008 to 2016 were provided from the Department of Dermatology, Paracelsus Medical University, Salzburg, Austria as well as from the Department of Dermatology, University Hospital of Zurich, Zurich, Switzerland [34]. This study was performed according to the principles expressed in the declaration of Helsinki. Clinical data of MM patients and the composition of immune cell infiltration of the respective tumor lesions were available [34].

### Flow cytometry

Cell surface associated protein levels, proliferation rate and frequency of apoptosis were analyzed in human melanoma cell lines by flow cytometry with the MACSQuant^®^ Analyzer 10 (Miltenyi Biotec, Bergisch Gladbach, Germany). Data analysis was performed using the Flowing Software (Cell Imaging Core, Turku Centre for Biotechnology, Finland). For CD44s detection, the mouse monoclonal antibody (mAb) CD44 (156-3C11) [FITC] (Bio-Techne, Wiesbaden, Germany) was utilized. Apoptosis was monitored with the Annexin-V-FITC Apoptosis-Detection-Kit (Beckman Coulter, Brea, CA, USA) following the manufacturer´s instructions.

### Cloning of miR expression vectors and cell transfection

The miR expression vectors were generated by cloning the miR gene with its flanking regions into the multiple cloning site of the pmR-m-Cherry vector (Clontech, Mountain View, CA, USA). After sequencing, the vectors were transiently transfected into human melanoma cell lines using Viromer^®^ RED (Lipocalyx, Halle, Germany). The BLM cells were transfected transiently. Due to the very low transfection efficacy of the WM1552C cells, these cells were transfected stably by geneticin supplemented medium (500 µg/ml).

### Cell biological assays

Cell proliferation, migration and apoptosis were determined in miR overexpressing cells 24 h after transfection. Briefly, 1 × 10^5^ transfected cells/well were seeded and grown in complete DMEM medium. The cell proliferation was assessed by determination of the total cell number after 24 h, 48 h, 72 h and 96 h of culture. For investigating chemotherapy sensitivity, cells were treated with 1 µg/ml daunorubicin (Sigma Aldrich, St.Louis, USA) for 2 h, washed three times with PBS and then cultured for additional 24 h prior to analysis.

An undirected migration assay was performed using the CytoSMART™ System (Lonza, Basel, Switzerland) by seeding 4 × 10^4^ cells 48 h after transient transfection into standard T75 cell culture flasks (Sarstedt, Nürmbrecht, Germany) with fresh medium. After 4 h adherence, the migration assays were started by recording a picture every 5 min for a total of 6 h. For data evaluation the software Tracking Tool™ PRO v2.0 (Gradientech, Uppsala, Sweden) was applied.

### Nucleic acid isolation, cDNA synthesis and qPCR

DNA was isolated from different human cell lines using the NucleoSpin^®^ Tissue kit (Macherey-Nagel, Düren, Germany) according to the manufacturer`s instructions. Total RNA was extracted from non-affected human skin tissue samples derived from formalin fixed human body donors (gluteal region) with peqGold TriFast (VWR International, Darmstadt, Germany) according to the manufacturer’s instructions followed by RQ1 RNase-free DNase I treatment (Promega, Mannheim, Germany). For miR-specific cDNA synthesis, 1 µg of total cellular RNA was reverse transcribed with miR-specific stem loop primers [35, 36] and the RevertAidTM H Minus reverse transcriptase (Thermo-Fisher Scientific, Waltham, USA).

For qPCR target-specific primers and the iQ™ SYBR^®^ Green Supermix (Biorad, Hercules, USA) were applied. Subsequently, the reactions were run in a qTOWER^3^ G (Analytik Jena, Jena, Germany). Absolute copy numbers were measured against an external miR-specific TOPO-TA plasmid standard (Invitrogen, Carlsbad, CA, USA) containing the respective PCR product [37, 38]. Reactions were performed in triplicates of biological replicates and results are described as mean values with standard deviation and *t*-test. All oligonucleotides used are listed in Table 5.

**Table 4:** List of the applied antibodies

name	method	dilution	species	manufacturer
anti-CD44s (156-3C11) mAb	IHC, WB	1:1000	mouse	Thermo-Fisher Scientific
HRP-linked anti-Mouse	IHC	-	goat	DAKO
HRP-linked anti-mouse	WB	1:1000	goat	dianova
anti-GAPDH (14C10) mAb	WB	1:12000	rabbit	Cell Signaling
HRP-linked anti-rabbit	WB	1:12000	goat	Cell Signaling
anti-CD44 (156-3C11) FITC mAb	FACS		mouse	biotechne

**Table 5:** List of the applied oligonucleotides

name	application	sequence (5′ → 3′)	condition
Klon3UTR-#A fw	cloning	GTGTAACACCTACACCATTATC	60°C
Klon3UTR-#A rev	cloning	GCAAAGCCTTTCACAGGAGAG	60°C
Klon3UTR-#B fw	cloning	CCTGTCCTGGAATCAGAGTTG	60°C
Klon3UTR-#B rev	cloning	TTGGTGTTGTTATGAATCTC	60°C
Kl3UTRHLAGfw	cloning	AAACAGCTGCCCTGTGT	60°C
Kl3UTRHLAGev	cloning	AAAGTTCTCATGTCTTCCATTT	60°C
708-5pRT-Rct	stem-loop primer	GTCGTATCCAGTGCAGGGTCCGAGGTATTCGCACTGGATACGACCCCAGC	42°C
708-5p PCR fw	qPCR	GCCCAAGGAGCTTACAATCTA	60°C
671-5pRT-Rct	stem-loop primer	GTCGTATCCAGTGCAGGGTCCGAGGTATTCGCACTGGATACGACCTCCAG	42°C
671-5p PCR fw	qPCR	GCCCAGGAAGCCCTGGAGGGG	60°C
608RT-PCR	stem-loop primer	GTCGTATCCAGTGCAGGGTCCGAGGTATTCGCACTGGATACGACACGGAG	42°C
608 PCR fw	qPCR	GCCCAGGGGTGGTGTTGGGACAG	60°C
541RT-Rct	stem-loop primer	GTCGTATCCAGTGCAGGGTCCGAGGTATTCGCACTGGATACGACAGTCCA	42°C
541 PCR fw	qPCR	GCCCTGGTGGGCACAGAATC	60°C
520c-3pRT-Rct	stem-loop primer	GTCGTATCCAGTGCAGGGTCCGAGGTATTCGCACTGGATACGACACCCTC	42°C
520c-3p PCR fw	qPCR	GCCCAAAGTGCTTCCTTTTA	60°C
512-3pRT-Rct	stem-loop primer	GTCGTATCCAGTGCAGGGTCCGAGGTATTCGCACTGGATACGACGACCTC	42°C
512-3p PCR fw	qPCR	GCCCAAGTGCTGTCATAGCT	60°C
491-5pRT-Rct	stem-loop primer	GTCGTATCCAGTGCAGGGTCCGAGGTATTCGCACTGGATACGACCCTCAT	42°C
491-5p PCR fw	qPCR	GCCCAGTGGGGAACCCTTCC	60°C
373-3pRT-Rct	stem-loop primer	GTCGTATCCAGTGCAGGGTCCGAGGTATTCGCACTGGATACGACACACCC	42°C
373-3p PCR fw	qPCR	GCCCGAAGTGCTTCGATTTTG	60°C
330-3pRT-Rct	stem-loop primer	GTCGTATCCAGTGCAGGGTCCGAGGTATTCGCACTGGATACGACTCTCTG	42°C
330-3p PCR fw	qPCR	GCCCGCAAAGCACACGGCCTG	60°C
328-3pRT-Rct	stem-loop primer	GTCGTATCCAGTGCAGGGTCCGAGGTATTCGCACTGGATACGACACGGAA	42°C
328-3p PCR fw	qPCR	GCCCCTGGCCCTCTCTGCCC	60°C
216a-5pRT-Rct	stem-loop primer	GTCGTATCCAGTGCAGGGTCCGAGGTATTCGCACTGGATACGACTCACAG	42°C
216a-5p PCR fw	qPCR	GCCCTAATCTCAGCTGGCAA	60°C
199a-3pRT-Rct	stem-loop primer	GTCGTATCCAGTGCAGGGTCCGAGGTATTCGCACTGGATACGACTAACCA	42°C
199a-3p PCR fw	qPCR	GCCCACAGTAGTCTGCACAT	60°C
152RT-Rct	stem-loop primer	GTCGTATCCAGTGCAGGGTCCGAGGTATTCGCACTGGATACGACCCAAGT	42°C
152 PCR fw	qPCR	GCCCTCAGTGCATGACAGA	60°C
143-3pRT-Rct	stem-loop primer	GTCGTATCCAGTGCAGGGTCCGAGGTATTCGCACTGGATACGACGAGCTA	42°C
143-3p PCR fw	qPCR	GCCCTGAGATGAAGCACTG	60°C
34a-5pRT-Rct	stem-loop primer	GTCGTATCCAGTGCAGGGTCCGAGGTATTCGCACTGGATACGACACAACC	42°C
34a-5p PCR fw	qPCR	GCCCTGGCAGTGTCTTAGCT	60°C
stem loop reverse primer	qPCR	GTGCAGGGTCCGAGGT	60°C
Klon2miR-34a fw	cloning	AAACTCGAGgagacagttgctgaaggt	60°C
Klon2miR-34a rev	cloning	AAAGGATCCctgcaagacggggttaat	60°C
Klon2miR-143 fw	cloning	AAACTCGAGaaactggtctgcccaggacta	60°C
Klon2miR-143 rev	cloning	AAAGGATCCtgggtgatggagtgagactgt	60°C
invitroHLA-Gfw	PCR	CCCCCCTAATACGACTCACTATAGGGAAAAAACAGCTGCCCTGTGT	60°C
invitroHLA-Grev	PCR	TTTTTTTTTTTTTTTTTTTTTTTTTTTTTTTTTTTAAAGTTCTCATGTCTTCCATTT	60°C
ivCD443Afw	PCR	CCCCCCTAATACGACTCACTATAGGGAAAGTGTAACACCTACACCATTATC	60°C
ivCD443Arev	PCR	TTTTTTTTTTTTTTTTTTTTTTTTTTTTTTTTTTTGCAAAGCCTTTCACAGGAGAG	60°C
ivCD443Bfw	PCR	CCCCCCTAATACGACTCACTATAGGGAAACCTGTCCTGGAATCAGAGTTG	60°C
ivCD443Crev4	PCR	TTTTTTTTTTTTTTTTTTTTTTTTTTTTTTTTTTTTTGGTGTTGTTATGAATCTC	60°C

### Western blot

Proteins of transiently transfected cells were extracted 48 h after transfection as recently described [50]. 30 µg of total protein/lane were loaded on Novex™ 4–20% Tris-Glycine Mini Gels, WedgeWell™ (Invitrogen, Carlsbad, CA, USA). After electrophoresis, the separated proteins were blotted onto a 0.2 μm nitrocellulose membrane (GE Healthcare, Little Chalfont, UK) for 2 h at 150 mA/gel. The blots were then blocked for 2 h at room temperature followed by incubation with primary antibodies over night at 4°C. After 5 washing steps the blots were incubated with the secondary antibodies and bands were visualized with the Amersham ECL Prime Western Blotting Detection reagent (Amersham, Chalfont St Giles, UK) on a ChemiDoc Touch Imaging system (Biorad, Hercules, USA). All applied antibodies used are listed in the Table 4.

### Immunhistochemistry

Standard immunohistochemistry was performed to assess the CD44 protein expression in the diverse formalin fixed human skin tissues. Briefly, 5 µg sections of paraffin embedded blocks were deparaffinized and hydrated prior to epitope retrieval treatment with HIER citrate buffer (Zytomed Systems, Berlin, Germany) for 20 min at 95°C. Endogenous peroxidases were blocked by incubation with 1% (V/V) H_2_O_2_ for 20 min at room temperature, before serum (Agilent, Santa Clara, USA) for 1 h at room temperature and incubated with the primary CD44 antibody overnight at 4°C. Negative controls were incubated solely in TBS-T. After washing steps, the HRP-linked secondary antibody was added. The detection was performed with the DAB liquid kit (Dako, Santa Clara, USA) and finally the reaction was quenched by immersion in tap water. After haematoxylin staining and dehydration, the slices were mounted with Eukitt^®^ mounting medium (ORSAtec, Bobingen, Germany). All applied antibodies used are listed in the Table 4. The photo documentation was performed with a DMi8 microscope (Leica, Wetzlar, Germany). The CD44s expression was scored by two researchers (J.F. and S.J.B.) as follows: 1 – weak, 2 – medium, and 3 – strong.

### Modified miR enrichment assay

To enrich CD44-specific miRs, the miTRAP technology [39] was modified. Briefly, the 3‘ UTR of CD44 was splitted into two overlapping fragments (#A, #B), which were in addition to the 3‘ UTR of HLA-G as an internal positive control amplified by PCR (Taq DNA Polymerase, Thermo Fisher Scientific) from genomic DNA and cloned into a TOPO™ TA Cloning™ Vector (Thermo Fisher Scientific). After sequencing of the respective 3’ UTRs, they were again amplified with specific primers containing the T7 promoter sequence within the forward primer for further *in vitro* transcription and the signal for a later poly(A) tail after *in vitro* transcription in the reverse primer. The resulting PCR products were purified by agarose gel electrophoresis. Subsequently, the purified PCR products were applied as templates for *in vitro* transcription over night using the T7 RiboMAX™ Kit (Promega, Madison, WI, USA). After DNase I digestion (Promega, Madison, WI, USA) the *in vitro* transcribed RNA constructs were purified with the MEGAclear Transcription Clean-Up Kit (Thermo Fisher Scientific) according to the manual.

The miR purification assay was performed in analogy to the previously published miTRAP assay with modification [37]. Instead of the fusion protein consisting of a MS2 loop and maltose binding protein in combination with amylose beads, the *in vitro* transcribed 3’ UTRs serving as baits for miR enrichment were polyadenylated and then linked to magnetic Dynabeads™ Oligo(dT)25 (Thermo Fisher Scientific). Furthermore, any containing polyadenylated RNA species, e.g. mature mRNAs, present in the applied cell lysate used as input were excluded prior usage by size exclusion columns (NucleoSpin^®^ miRNA, MACHEREY-NAGEL, Dürren, Germany). The principle of this assay is summarized in Figure 1A.

### Statistical analyses

Microsoft Excel 2016 (Microsoft Corporation, Albuquerque, USA) and OriginPro 2017 (OriginLab, Northampton, USA) were used for calculation of mean, standard deviation and students’ test under assumption of unequal variance. Data were significant with *p*-values of *p* < 0.05 and marked as follows: ^*^ *p* < 0.05, ^**^ *p* < 0.01, ^***^ *p* < 0.001.

## Conclusions

The tumor suppressive miR-143-3p was identified as the most potent CD44 inhibitory miR, which affects growth characteristics of melanoma cells suggesting the implementation of miR-143-3p as as a potential anti-CD44 therapy of malignant melanoma.

## ^Abbreviations^

CD: cluster of differentiation
CD44s: “standard” CD44 isoform
CD44v: “variant” isoform
CDS: coding sequence
CSC: cancer stem cell
ECM: extracellular matrix
FITC: fluorescein isothiocyanate
HA: hyaluronic acid
HRP: horseradish peroxidase
IHC: immunohistochemistry
mAb: monoclonal antibody
MM: malignant melanoma
miR: microRNA
MM: malignant melanoma
n.d: not detectable
n.r: not reported
OS: overall survival
PI: propidium iodide
UTR: untranslated region
